# Effect of subinhibitory concentrations of tigecycline and ciprofloxacin on the expression of biofilm-associated genes and biofilm structure of *Staphylococcus epidermidis*

**DOI:** 10.1099/mic.0.000453

**Published:** 2017-05-09

**Authors:** Ewa Szczuka, Lucyna Jabłońska, Adam Kaznowski

**Affiliations:** Department of Microbiology, Institute of Experimental Biology, Faculty of Biology, Adam Mickiewicz University, Poznań, Poland

**Keywords:** *Staphylococcus epidermids*, subinhibitory concentration of antibiotics, gene expression, biofilm formation

## Abstract

*Staphylococcus epidermidis* is a leading cause of foreign body-associated infections. This is related to the bacterium's ability to form biofilms on synthetic materials. Bacteria within a biofilm may be exposed to subinhibitory concentrations (sub-MICs) of antibiotics because of an agent's limited penetration into the biofilm core. Here, we investigated the effect of sub-MICs of tigecycline and ciprofloxacin on the expression of biofilm-associated genes, i.e. *icaA, altE* and *sigB,* and the biofilm structure of five clinical isolates of *S. epidermidis*. For most tested isolates, the expression of these genes increased after exposure to 0.25 MIC and 0.5 MIC tigecycline. A slight decrease in *icaA*mRNA levels was observed only in two isolates in the presence of 0.25 MIC tigecycline. The effect of ciprofloxacin exposure was isolate-dependent. At 0.5 MIC, ciprofloxacin induced an increase of *sigB* and *icaA*mRNA levels in three of the five tested isolates. At the same time, expression of the *altE* gene increased in all isolates (from 1.3-fold to 42-fold, depending on the strain). Confocal laser scanning microscopy analysis indicated that sub-MIC ciprofloxacin decreased biofilm formation, whereas tigecycline stimulated this process. Our data suggest that sub-MIC tigecycline may have bearing on the outcome of infections.

## Introduction

*Staphylococcus epidermidis* is the major cause of catheter-related bloodstream infections and other infections associated with implanted medical devices. Its pathogenesis is associated with the ability to attach to biomaterials and to develop a biofilm [1–6]. One autolysin protein, AtlE, mediates the primary attachment of *S. epidermidis* cells to a polystyrene surface; it is also involved in the adhesion to vitronectin, an extracellular matrix protein which encapsulates devices after implantation into the human body [7]. The most common molecule used by *S. epidermidis* strains for intercellular adhesion and cell accumulation is polysaccharide intercellular adhesion (PIA), encoded by an *icaADBC* operon [5, 8, 9]. The expression of an *ica* (intercellular adhesion gene) locus is regulated by the alternative sigma factor σ^B^, which is encoded by four genes: *rsbU, rsbV*, *rsbW* (gene encoding the σ^B^ regulator) and *sigB* (gene encoding σ^B^). The *rsbU* and *rsbV* gene products are responsible for stimulating *sigB* activity, whereas the *rsbW* gene product is a negative regulator. Synthesis of σ^B^ indirectly represses the transcription of the *icaR* gene, a negative regulator of *icaADBC* transcription, and therefore allows biofilm formation [10].

Bacteria inside biofilms can be up to 1000 times more tolerant to antimicrobial agents than their planktonic counterparts, which may hinder eradication of biofilm-associated infections [6]. Tigecycline, a glycylcycline antibiotic, is highly active *in vitro* against bacteria within a biofilm [11, 12]. Tigecycline acts by inhibiting protein translation in bacteria, mediated by binding to the 30S ribosomal subunit and by blocking the association of charged tRNA to the A site of the ribosome [13]. Animal model data indicate efficient eradication of staphylococcal biofilms by tigecycline [14]. Fluoroquinolones, including ciprofloxacin, may also be useful in the treatment of biofilm-associated infections, especially when high doses of the drug are employed [15]. Ciprofloxacin acts through inhibition of DNA gyrase and topoisomerase IV activity and inhibition of DNA synthesis [16].

It is likely that cells within a biofilm are exposed to subinhibitory concentrations (sub-MICs) of antibiotics during antibiotic chemotherapy because of the limited penetration into the biofilm core [17, 18]. Several studies have demonstrated that sub-MIC antimicrobial agents can affect the expression of *S. aureus* virulence determinants, such as toxins, enzymes, regulatory proteins and adhesion, and other surface proteins [17–21]. For example, sub-MIC tigecycline reduces the expression of *tst* genes that encode toxic shock syndrome toxin 1 (TSST-1) leading to reduced toxin levels in *S. aureus* strains [17]. Expression of the *pvl* gene encoding Panton–Valentine leukocidin (PVL), a pore*-*forming toxin that can disrupt the host cell, is also reduced in the presence of sub-MIC tigecycline [21]. Expression of other virulence-associated genes, i.e. genes encoding enzymes involved in capsule synthesis, was also reduced in the presence of a sublethal concentration of tigecycline [17]. Much less is known about the effect of sub-MIC antibiotics on the expression of virulence-associated genes and the virulence potential of *S. epidermidis* strains.

This study was performed to investigate the effect of sub-MIC tigecycline and ciprofloxacin on the expression of the *icaA*, *altE* and *sigB* genes, and on biofilm structures of clinical *S. epidermidis* isolates.

## Methods

### Bacterial strains and antimicrobial agents

Five *ica*- and *altE-*positive biofilm-forming *S. epidermidis* isolates (MPU 75, MPU 51, MPU 52, MPU 57, MPU 85) were evaluated in this study. These isolates were isolated from patients with bloodstream infections and were stored as glycerol stocks at −80 °C. Tigecycline (Sigma-Aldrich) and ciprofloxacin (Sigma-Aldrich) powders were as provided by the manufacturer. MIC testing was performed by broth microdilution in Mueller–Hinton II broth according to the methods of the Clinical and Laboratory Standards Institute [22]. The tigecycline MIC values were as follows: 0.5 µg ml^−1^ for three isolates (MPU 52, MPU 57, MPU 85) and 0.125 µg ml^−1^ for two isolates (MPU 76 and MPU 51). The ciprofloxacin MIC values were as follows: 1 µg ml^−1^ for one isolate (MPU 52) and 4 µg ml^−1^ for four isolates (MPU 57, MPU 85, MPU 76, MPU 51). Cultures were incubated in the absence or presence of the antibiotics at 0.25 MIC (i.e. 0.125 or 0.03125 µg ml^−1^ for tigecycline and 0.250 or 1 µg ml^−1^ for ciprofloxacin) and 0.5 MIC (i.e. 0.250 or 0.0625 µg ml^−1^ for tigecycline and 0.5 or 2 µg ml^−1^ for ciprofloxacin) at 37 °C for 24 h.

### RNA extraction, cDNA synthesis and quantitative real-time PCR

The samples were pelleted by centrifugation and the bacterial pellets were suspended in 1 ml Trizol Reagent (Sigma). Cell walls were mechanically disrupted by vigorous vortexing after mixing with glass beads (425–600 µm diameter, Sigma). Then, 100 µl 1-bromo-3-chloropropane (INC) was added. The samples were incubated with periodical mixing for 30 min at 20 °C and centrifuged at 12 000 ***g*** for 15 min. The aqueous phase (450 µl) was transferred to a fresh microtube and mixed with isopropanol (500 µl) to precipitate RNA. After vigorous vortex mixing, the samples were centrifuged at 12 000 ***g*** for 10 min. The RNA pellets were washed with ice-cold 75 % ethanol to remove isopropanol and centrifuged at 12 000 ***g*** for 5 min. This step was repeated twice. Air-dried RNA samples were re-suspended in 25 µl of RNase-free water. RNA integrity was verified by analysing 5 µl of the total RNA samples by 1.5 % agarose gel electrophoresis in Tris-borate-EDTA (TBE) buffer. RNA concentration and purity were determined using the Nano-100 Micro-Spectrophotometer (Hangzhou Allsheng Instruments). cDNA was synthesized using the Thermo Scientific RevertAid First Strand cDNA Synthesis kit (Thermo Scientific) according to the manufacturer's instructions, with 1 µg of RNA as the template. Real-time amplification was performed with 500 ng cDNA, 10 µl SYBR GreenER qPCR SuperMix (Invitrogen) and 1 µl each of forward and reverse primers (200 nM each). The reaction volume was adjusted to 20 µl with RNase- and DNase-free water. Primer sequences for *icaA*, *altE*, *sigB* and rRNA were as published [23, 24]. Real-time PCR was performed using the CFX96 Real-Time System C1000 Touch (Bio-Rad, Hemel Hempstead, UK) and the following cycle parameters: 50 °C for 2 min and 95 °C for 10 min, followed by 40 cycles of 95 °C for 15 s and then 60 °C for 60 s. Samples for real-time RT-PCR were run in triplicate. The *rRNA* gene was used as an internal control to normalize the levels of expression between samples. Real-time RT-PCR data were analysed by the 2^−^^∆∆*C*_t_^ method [25].

### Confocal laser scanning microscopy (CLSM)

Bacteria were grown overnight in Mueller–Hinton II broth supplemented with the appropriate antibiotic (at 0.25 MIC and 0.5 MIC) or without antibiotics in Lab-TekII cell-culture chamber wells (Nunc) [26]. After removal of the medium and gentle washing of the wells three times with PBS to remove planktonic cells, the adherent cells were stained using SYTO stain and propidium iodide (Live/Dead BacLight Bacterial Viability kits; Invitrogen) for 15 min and observed by fluorescence microscopy (Carl Zeiss LSM 510/Axioveut 200M). Carl Zeiss confocal software and the computer program comstat were used to analyse the three-dimensional biofilm images and for data analysis [27, 28]. For every sample, three microscopic fields were analysed and the means were calculated.

## Results and discussion

We examined the effect of sub-MIC tigecycline and ciprofloxacin, two antibiotics used to treat staphylococcal infections, on the expression of genes encoding proteins that contribute to the pathogenicity of *S. epidermidis* and on the overall biofilm structure of clinical bacterial isolates.

### Effect of sub-MIC tigecycline and ciprofloxacin on expression of the *S. epidermidis icaA* gene

The *icaA* gene encodes a transmembrane protein with homology to *N*-acetyl-glucosaminyl transferases that synthesize PIA [5]. Increased *icaA* expression can stimulate the production of PIA, which can lead to increased intercellular adhesion of bacterial cells in a biofilm [23]. Recently, Gomes *et al.* [24] reported enhanced *icaA* expression in *S. aureus* strains exposed to rifampicin at sub-MIC as well as rifampicin in combination with gentamicin or clindamycin. Tetracycline, quinupristin-dalfopristin and erythromycin at sub-MICs were also found to stimulate *icaA* expression [23, 29]. In contrast, subinhibitory concentrations of penicillin, oxacillin, chloramphenicol, clindamycin, gentamicin, ofloxacin, vancomycin and teicoplanin had no effect on the expression of *icaA* [29].

**Fig. 1.**
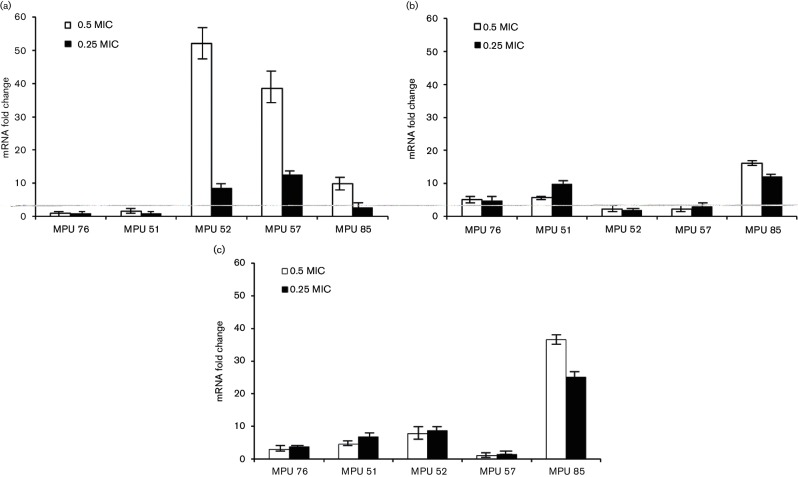
Effect of tigecycline on the expression of *icaA* (a), *altE* (b), and *sigB* (c) in *S. epidermidis* isolates MPU 75, MPU 51, MPU 52, MPU 57 and MPU 85. The relative expression of *icaA*, *altE* and *sigB* in antibiotic-exposed strains is plotted in comparison to that in unexposed controls, with rRNA as a reference gene. Real-time RT-PCR data were analysed by the 2^−^^∆∆*C*_t_^ method. Samples for real-time RT-PCR were run in triplicate. Error bars represent standard deviation.

The expression of *icaA* was increased by onefold to 52-fold when the isolates were grown in the presence of 0.5 MIC tigecycline (Fig. 1). Tigecycline at 0.25 MIC resulted in an increase in *icaA* mRNA levels (by 2.6-fold to 12.6-fold) in three of the five isolates tested, namely, MPU 52, MPU 57 and MPU 85. Under these conditions, *icaA* expression was slightly decreased (0.92-fold and 0.95-fold) in isolates MPU 76 and MPU 51. Tigecycline has previously been shown to reduce the expression of *icaC*, which encodes IcaC, the protein involved in the cytoplasmic membrane export of the extracellular poly-N-acetylglucosamine (PNAG) in *S. aureus* strains [17].

**Fig. 2.**
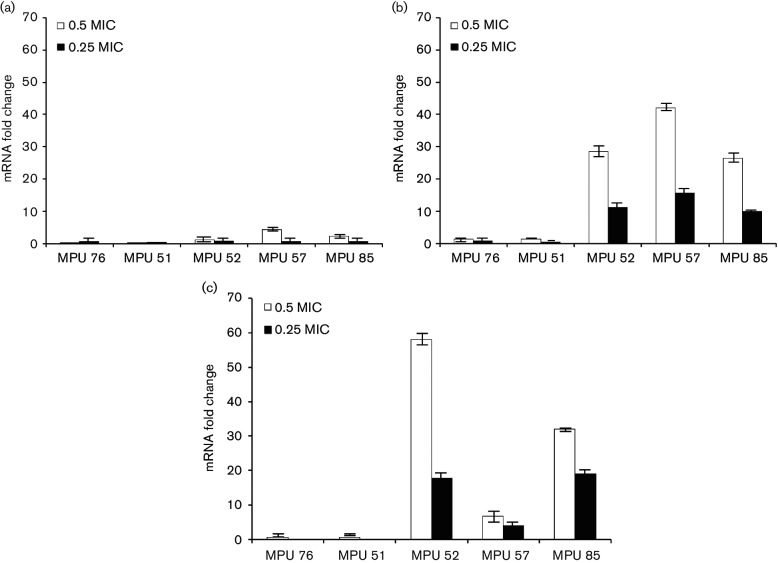
Effect of ciprofloxacin on the expression of *icaA* (a)*, altE* (b), and *sigB* (c) in *S. epidermidis* isolates MPU 75, MPU 51, MPU 52, MPU 57 and MPU 85. The relative expression of *icaA*, *altE* and *sigB* in antibiotic-exposed strains is plotted in comparison to that in unexposed controls with rRNA as a reference gene. Real-time RT-PCR data were analysed by the 2^−^^∆∆*C*_t_^ method. Samples for real-time RT-PCR were run in triplicate. Error bars represent standard deviation.

As shown in Fig. 2, the effect of ciprofloxacin on *icaA* expression was different in different isolates. For three isolates (MPU 52, MPU 57 and MPU 85), exposure to ciprofloxacin at 0.5 MIC increased *icaA* gene expression by 1.2-fold to 4.3-fold. Slightly decreased *icaA* mRNA levels were observed in the remaining clinical isolates (MPU 76 and MPU 51) exposed to 0.5 MIC of the antibiotic. In contrast, exposure to ciprofloxacin at 0.25 MIC resulted in decreased *icaA* expression in all isolates.

### Effect of sub-MIC tigecycline and ciprofloxacin on expression of the *S. epidermidis altE* gene

The *altE* gene encodes AtlE autolysin, which mediates the adhesion of bacteria to vitronectin and their initial attachment to polystyrene surfaces [7]. Expression of *altE* was increased when the *S. epidermidis* isolates were grown in the presence of both 0.25 and 0.5 MIC tigecycline (by 1.9-fold to 11.9-fold and by 2.2-fold to 16.1-fold, respectively). Previously, it was shown that sub-MIC tigecycline increased the expression of *S. aureus* genes encoding adhesion molecules, such as *cna* (encoding collagen-binding protein), *clfB* (encoding fibrinogen-binding protein) and *fnbA* (encoding fibronectin-binding protein) [17]. Increased expression of adhesin-encoding genes may result in more efficient microbial adhesion to biological and abiotic surfaces, which is considered the first, and probably the most crucial, step in the establishment of infection.

As shown in Fig. 2, exposure to 0.5 MIC ciprofloxacin led to increased *altE* mRNA levels in all tested isolates (from 1.3-fold to 42-fold, depending on the isolates). Additionally, increased *altE* gene expression (by 9.9-fold to 15.6-fold) was observed in MPU 52, MPU 57 and MPU 85 clinical isolates upon exposure to ciprofloxacin at 0.25 MIC. Decreased *altE* gene expression was observed in MPU 76 and MPU 51 cells grown in the presence of 0.25 MIC ciprofloxacin.

### Effect of sub-MIC tigecycline and ciprofloxacin on expression of the *S. epidermidis sigB* gene

Tigecycline at sub-MIC concentrations led to increased *sigB* mRNA levels in all tested isolates. Expression of *sigB* was increased by >twofold in isolates MPU 76, MPU 51, MPU 52 and MPU 85 and expression of *sigB* increased by 1.1-fold in MPU 57 in response to 0.5 MIC tigecycline. Exposure to tigecycline at 0.25 MIC resulted in increased *sigB* expression in four isolates (>twofold); expression of *sigB* increased to a lesser extent in MPU 57 (1.3-fold). Ciprofloxacin exposure (0.5 MIC) resulted in decreased *sigB* expression in two isolates (MPU 76 and MPU 51). Overall, with the exception of isolates MPU 52 and MPU 57, exposure to sub-MIC tigecycline increased *S. epidermidis sigB* gene expression to a greater extent than sub-MIC ciprofloxacin treatment. Previous studies demonstrated the role of the *sigB* gene in biofilm stability [30]. However, *sigB* status does not affect the primary attachment of cells to plastic and the early phase of biofilm development [30, 31]. Therefore, it may be speculated that increased *sigB* expression may improve the structural integrity of a biofilm. Subsequently, the biofilm might become more resistant to physical forces, such as shear forces produced by blood flow. This might enable the establishment of infection and infection persistence in the host.

### Effect of sub-MIC tigecycline and ciprofloxacin on the structure of *S. epidermidis* biofilms

Following the gene expression analyses, we investigated the effect of sub-MIC tigecycline and ciprofloxacin on *S. epidermidis* biofilm structure *in vitro.* CLSM was used to visualize the biofilm structure, and the comstat program was used to analyse physical biofilm parameters, i.e. biofilm thickness, the area occupied by bacterial layers, biomass (i.e. biomass volume divided by the area of view), surface area-to-biovolume ratio and roughness coefficient (an indicator of biofilm heterogeneity).

The number of adherent bacteria was reduced in the presence of 0.5 MIC ciprofloxacin with changes in the overall biofilm morphology, thickness, substratum coverage and roughness (Table 1). For example, the MPU 76 isolate exposed to ciprofloxacin formed a biofilm that was structured differently compared to that of the untreated control, i.e. the biofilm was composed of individual clusters of bacteria widely dispersed on the glass coverslip surface. The effect of ciprofloxacin was concentration-dependent, i.e. a lower number of adherent cells was observed in the presence of 0.5 MIC than in the presence of 0.25 MIC ciprofloxacin. Similarly to our results, Yassien and Khardori [15] reported that sub-MIC ciprofloxacin inhibits the adhesion of bacteria to vascular catheters, leading to reduced biofilm density. In contrast, Haddadin *et al*. [32] demonstrated neither an inhibitory nor a stimulatory effect of ciprofloxacin on biofilm formation by *S. aureus* strains. This discrepancy may be explained by the inhibitory effect of sub-MIC ciprofloxacin on bacteria and subsequent biofilm growth. A significant reduction in c.f.u. in the presence of 0.5 MIC ciprofloxacin was demonstrated previously [15].

**Table 1.** Effect of sub-MIC tigecycline (TIG) and ciprofloxacin (CIP) on the properties of biofilms formed by *S. epidermidis* isolates The comstat program was used to analyse physical biofilm parameters, i.e. biofilm thickness, the area occupied by bacterial layers, biomass (i.e. biomass volume divided by the area of view), surface area-to-biovolume ratio and roughness coefficient (an indicator of biofilm heterogeneity. For every sample, three microscopic fields were analysed and the means were calculated. In the table we show only the *P* value for this test.

	Treatment	Strain MPU 52	Strain MPU 85	Strain MPU 76	Strain MPU 57	Strain MPU 51
Average thickness (µm)*	No antibiotics	28±2	30±2	29±1	29±2	31±2
	TIG. 0.5 MIC	38±6 (*P*=0.0076)¶	44±3 (*P*=0.00016)	36±3 (*P*=0.0031)	39±6 (*P*=0.010)	39±4 (*P*=0.0028)
	TIG. 0.25 MIC	34±4 (*P*=0.017)	40±7 (*P*=0.0080)	31±2 (*P*=0.18)	30±1 (*P*=1)	33±3 (*P*=0.189)
	CIP. 0.5 MIC	19±2 (*P*=0.0007)	20±2 (*P*=0.00045)	22±4 (*P*=0.0070)	18±3 (*P*=0.00092)	24±1 (*P*=0.00038)
	CIP. 0.25 MIC	23±4 (*P*=0.0010)	27±3 (*P*=0.06)	26±1 (*P*=0.0031)	21±3 (*P*=0.0030)	30±2 (*P*=0.37)
Biomass (µm^3^/µm^2^)†	No antibiotics	26.62±3.32	25.08±2.00	22.70±1.75	21.29±2.18	24.95±0.91
	TIG. 0.5 MIC	31.55±5.78 (*P*=0.06)	35.65±1.33 (*P*=0.0001)	28.91±1.18 (*P*=0.0007)	28.30±1.01 (*P*=0.01)	34.06±7.54 (*P*=0.017)
	TIG. 0.25 MIC	27.37±5.97 (*P*=0.76)	24.78±7.35 (*P*=0.90)	25.22±1.54 (*P*=0.024)	22.38±3.04 (*P*=0.60)	27.32±4.07 (*P*=0.012)
	CIP. 0.5 MIC	16.95±5.97 (*P*=0.011)	15.34±8.97 (*P*=0.03)	12.31±4.29 (*P*=0.001)	8.16±1.77 (*P*=0.001)	20.28±0.93 (*P*=0.0004)
	CIP. 0.25 MIC	17.96±8.22 (*P*=0.032)	21.98±5.97 (*P*=0.178)	16.63±1.42 (*P*=0.00094)	7.68±1.08 (*P*=0.001)	25.49±2.89 (*P*=0.057)
Substratum coverage (%)‡	No antibiotics	96±3	99±0	98±1	96±2	98±0
	TIG. 0.5 MIC	96±2 (*P*=0.77)	99±0 (*P*=1)	99±0 (*P*=0.11)	96±3 (*P*=0.77)	99±0 (*P*=1)
	TIG. 0.25 MIC	98±1 (*P*=0.007)	98±1 (*P*=0.11)	99±0 (*P*=0.11)	98±1 (*P*=0.089)	99±0 (*P*=1)
	CIP. 0.5 MIC	92±1 (*P*=0.77)	94±4 (*P*=0.08)	67±21 (*P*=0.014)	70±18 (*P*=0.007)	97±1 (*P*=0.11)
	CIP. 0.25 MIC	96±5 (*P*=0.015)	98±1 (*P*=0.11)	92±4 (*P*=0.015)	72±11 (*P*=0.001)	99±0 (*P*=1)
Roughness§	No antibiotics	0.082±0.021	0.076±0.008	0.097±0.007	0.136±0.005	0.099±0.019
	TIG. 0.5 MIC	0.080±0.004 (*P*=0.765)	0.077±0.006 (*P*=0.605)	0.080±0.024 (*P*=0.085)	0.110±0.001 (*P*=0.000070)	0.120±0.013 (*P*=0.03)
	TIG. 0.25 MIC	0.098±0.032 (*P*=0.259)	0.124±0.022 (*P*=0.002)	0.103±0.007 (*P*=0.101)	0.092±0.005 (*P*=0.000030)	0.120±0.011 (*P*=0.029)
	CIP. 0.5 MIC	0.119±0.048 (*P*=0.072)	0.118±0.041 (*P*=0.024)	0.225±0.042 (*P*=0.00072)	0.205±0.064 (*P*=0.026)	0.109±0.013 (*P*=0.234)
	CIP. 0.25 MIC	0.094±0.038 (*P*=0.421)	0.065±0.018 (*P*=0.14)	0.112±0.005 (*P*=0.0054)	0.230±0.003 (*P*=0.000000001)	0.088±0.023 (*P*=0.310)
Surface area-to- biovolume ratio (µm^2^/µm^3^)||	No antibiotics	4.09±0.96	4.18±0.21	5.18±0.35	5.97±0.94	5.03±1.11
	TIG. 0.5 MIC	4.02±1.04 (*P*=0.875)	4.04±0.22 (*P*=0.214)	4.46±0.39 (*P*=0.011)	5.90±1.76 (*P*=0.931)	3.55±0.32 (*P*=0.011)
	TIG. 0.25 MIC	4.53±0.91 (*P*=0.337)	5.29±0.46 (*P*=0.064)	4.83±1.09 (*P*=0.366)	5.26±0.63 (*P*=0.099)	4.28±0.51 (*P*=0.100)
	CIP. 0.5 MIC	4.59±2.15 (*P*=0.519)	4.94±1.46 (*P*=0.171)	10.35±2.39 (*P*=0.002)	12.25±2.88 (*P*=0.0024)	5.17±0.23 (*P*=0.697)
	CIP. 0.25 MIC	4.99±0.91 (*P*=0.121)	4.90±0.71 (*P*=0.032)	7.74±0.54 (*P*=0.0002)	15.6±1.61 (*P*=0.000059)	4.69±0.66 (*P*=0.388)

*Thickness of biofilm. Values are data from image stocks.

†Mean value of biofilm biomass.

‡Percentage of the area occupied by bacterial layers. 100 % area was defined as when all of the visual field was covered by bacterial biofilm.

§Mean value of roughness coefficient, which is an indicator of biofilm heterogeneity.

||Mean value of surface area-to-biovolume ratio.

¶The differences between control (without antibiotic) groups and experiments (groups) was tested by *t*-test.

*P*-Value of standard deviation.

Exposure of *S. epidermidis* isolates to 0.5 MIC tigecycline resulted in a more compact three-dimensional biofilm structure, covering most of the glass coverslip surface, compared to antibiotic-untreated biofilm. We observed a change in the bacterial count and in the thickness of the biofilm formed in the presence of sub-MIC tigecycline.

This study clearly demonstrates that tigecycline and ciprofloxacin, with different antimicrobial modes of action, also affect bacteria in biofilm differently.

Our study documents the different effects of sub-MIC tigecycline and ciprofloxacin on the expression of the *icaA*, *altE* and *sigB* genes in clinical *S. epidermidis* isolates. The effect of ciprofloxacin appeared to be isolate-dependent. Generally, tigecycline treatment (both at 0.25 and 0.5 MIC) led to increased expression of the *icaA*, *altE and sigB* genes in the isolates. The only exception was isolates MPU 51 and MPU 76, where *icaA* mRNA levels were slightly decreased upon exposure to 0.25 MIC tigecycline. Sub-MIC tigecycline also affected biofilm architecture. These data suggest that sub-MIC tigecycline may alter the pathogenesis of staphylococcal infections. In contrast, ciprofloxacin, at levels below the MIC for bacteria, may still display anti-staphylococcal biofilm activity, thereby limiting the progression of staphylococcal disease.

Our study provides insight into the response of *S. epidermidis* to antimicrobial agents employed at sub-MICs or in situations where antimicrobial agents cannot achieve their MIC, e.g. when the bacteria are encased in a biofilm with decreased drug concentration in the biofilm core.
